# Serum ferritin as an activity marker for granulamotosis with polyangiitis

**DOI:** 10.1080/0886022X.2017.1349675

**Published:** 2017-07-25

**Authors:** Hamit Kucuk, Ozkan Varan, Berna Goker, Berivan Bitik, Mehmet Akif Ozturk, Seminur Haznedaroglu, Abdurrahman Tufan

**Affiliations:** Department of Internal Medicine, Division of Rheumatology, Faculty of Medicine, Gazi University, Ankara, Turkey

**Keywords:** Granulomatosis with polyangiitis, ferritin, biomarker, activity, vasculitis

## Abstract

**Background:** Serum ferritin correlates well with the activities of systemic lupus erythematosus (SLE) and dermatomyositis, but it has not been previously studied in patients with vasculitis.

**Methods:** Medical records of granulomatosis with polyangiitis (GPA, Wegener’s granulomatosis) patients with at least six months of regular follow-up were evaluated. The activity of GPA was assessed with Birmingham Vasculitis Activity Score for Wegener’s Granulomatosis (BVAS/WG). Serum ferritin and other acute phase markers were measured at initial presentation.

**Results:** Serum ferritin levels were found to be the highest in GPA patients with alveolar hemorrhage, median (IQR) 1041 (1281) μg/L. Patients with renal disease also had high levels of ferritin and it was correlated with concurrent glomerular filtration rate (*r* = −0.65, *p* < .001). Serum ferritin is also correlated well with the BVAS/WG scores (*r* = 0.79, *p* < .001).

**Conclusions:** Measurement of serum ferritin might help in assessing disease activity of GPA.

## Introduction

Granulomatosis with polyangiitis (GPA, or Wegener’s granulomatosis) is a multi-system necrotizing vasculitis with high morbidity and mortality by disease itself and its intensive immunosuppressive treatment. GPA most commonly involves upper and lower airways and kidneys. Alveolar and gastrointestinal bleedings and other visceral involvements are primary causes of mortality. Current biomarkers are not helpful for the assessment of disease severity and reliable biomarkers are needed to guide its treatment [1–4].

Ferritin is a specific iron-binding protein which is primarily responsible for the storage of iron and protection of body from the potential toxicity of this element. Recent advances have shown that ferritin has roles beyond the storage of iron, such as modulation of inflammation, DNA binding and neuro-degeneration [5–7]. Production of ferritin is under tight control and several factors regulate its expression, including cytokines, growth factors, hormones, ischemia and oxidative stress [8].

Ferritin is a positive acute phase reactant; however, in certain inflammatory diseases like Still’s disease, it rises disproportionally high levels with respect to other acute-phase reactants, which suggest its possible contribution to the pathogenesis of disease during the active period of condition [9].

Role of serum ferritin as a marker of disease activity and involvement severity has already been shown in sepsis, graft rejection, dermatomyositis and SLE; however, it has not been previously studied in vasculitides [10–14]. Herein, we aim to investigate association of serum ferritin level with activity and severe manifestations of disease in patients with GPA.

## Methods

Medical records of GPA patients who were diagnosed at Gazi University Internal Medicine-Rheumatology Clinic between May 2010 and June 2015, and followed up at least 6 months were included. Those who died in the initial 6-month period of follow-up were also included.

All patients were classified as GPA according to the Chapel Hill Consensus Conference nomenclature [15]. Activity of GPA was assessed with Birmingham Vasculitis Activity Score for Wegener’s Granulomatosis (BVAS/WG) [16]. Glomerular filtration rate was calculated with MDRD formula [17]. Diagnosis of alveolar hemorrhage was suggested by chest X-ray or computed tomography and confirmed with bronchoscopy if there was no obvious hemoptysis. Patients with gastrointestinal involvement underwent comprehensive endoscopic examinations. Angiography and/or scintigraphy was performed when indicated. GPA was histopathologically confirmed in all patients with localized disease or renal involvement. Systemic/severe visceral (SV) involvement was defined as the presence of one of the severe visceral items of BVAS in a patient, when there was more than one visceral item, the patient was considered as multisystem visceral (MSV) involvement.

Patients with active infections which demonstrated with appropriate microbiologic examinations were excluded from the study. Other exclusion criteria were the presence of hematologic, hepatic or renal disorders and recent blood transfusions. Baseline erythrocyte sedimentation rate (ESR), C-reactive protein (CRP) and serum ferritin levels were retrieved from medical records. All of these parameters are among the admission routines of our clinic. Serum ferritin and acute phase reactants were determined at first presentation when the disease was active and patients were untreated yet.

SPSS windows v16.0 (SPSS Inc, Chicago, IL) was used for the analyses. Categorical and continuous variables of groups were compared with Chi-square and Mann–Whitney U-tests, respectively. Spearman’s test was used for correlation analyses. A *p* values ≤.05 was considered significant in all analyses. The ability of serum ferritin level in predicting systemic involvement and pulmonary hemorrhage are analyzed with receiver operating characteristics (ROC) analyses. When a significant cutoff value is observed, the sensitivity, specificity, positive and negative predictive values are presented. While evaluating the area under the curve (AUC), a 5% type-1 error level was used to accept a statistically significant predictive value of the test variables.

## Results

There were 42 patients with GPA in the study. Mean age of the study population was 49.0 ± 14.6 years and 24 (57.1%) patients were male. Except for a patient with rhinosinusitis and retro-orbital granuloma c-ANCA was positive in all patients which was determined with Immunofloresence Assay (IFA). Ten (23.8%) patients had localized or nonsevere manifestations, such as upper airway disease (Table 1). Thirty-two patients had systemic visceral involvement. Of these, 14 patients had alveolar hemorrhage and seven of them had required mechanical ventilation. Gastrointestinal (GI) involvement was observed in two patients. Fourteen patients had multi-system visceral disease and four had died within 6 months after the diagnosis.

**Table 1.** Disease manifestations of GPA patients in the study.

Disease manifestation	*n* (%)
Upper airways	23 (54.8)
Lung (cavities)	18 (42.9)
Alveolar hemorrhage	14 (33.3)
Glomerulonephritis	26 (61.9)
Mesenteric ischemia	2 (4.8)
Cochlear	7 (16.7)
Neuropathy	8 (19.0)
Local disease	10 (23.8)
Scleritis	2
Retro-orbital granuloma	2
Rhinosinusitis	5
Otitis (noncochlear)	1
Systemic visceral disease	18 (42.9)
Multisystem visceral disease	14 (33.3)

Erythrocyte sedimentation rate, CRP and serum ferritin levels were higher than reference values in all patients with systemic GPA. However, in localized/nonvisceral group, serum ferritin values were within normal limits except for a patient with retro-orbital granuloma (318 μg/L). There was no correlation between serum ferritin levels and other acute phase reactants, namely ESR and CRP in either localized or systemic disease. Serum ferritin correlated significantly with BVAS/WG (*r* = 0.79, *p* < .001). However, there was no correlation between BVAS/WG and other acute phase reactants.

**Figure 1.**
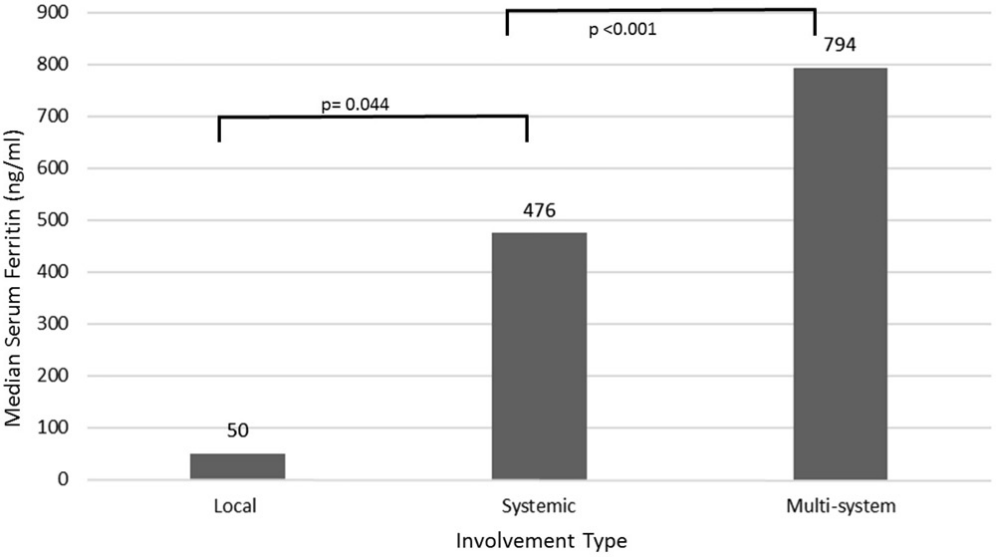
Serum ferritin levels according to visceral involvement. Visceral and multivisceral involvements had significantly higher ferritin levels compared to local/nonsevere involvements.

Patients with alveolar hemorrhage had the highest serum ferritin levels (Table 2). Patients with renal involvement also had high levels of ferritin and it was correlated well with the GFR (*r* = −0.65, *p* < .001). Finally, patients with MSV involvement had remarkably higher ferritin levels compared with SV group (*p* < .001) (Figure 1)

**Table 2.** Serum acute phase reactants and ferritin levels according to GPA manifestations.

	GPA manifestations				
Markers	Nonvisceral manifestations *n* = 10	Glomerulo-nephritis *n* = 26	Pulmonary hemorrhage *n* = 14	Multivisceral disease *n* = 14	Any visceral manifestation *n* = 32
ESR, mm/h	31 (28)	48 (73)	31 (46)	31 (60)	57 (57)
CRP, mg/l	9.3 (20)	30 (112)	59 (134)	36 (158)	48 (118)
Ferritin, μg/L	50 (73)	633 (1116)	1041 (1281)	794 (1317)	476 (1039)
BVAS/WG	2.5 (1.5)	10.5 (10)	15 (8.5)	14 (9.5)	9.5 (10.5)

Values are given as median (IQR).

BVAS/WG: Birmingham Vasculitis Activity Score for WG Vasculitis; CRP: C-reactive protein; ESR: erythrocyte sedimentation rate; GPA: granulomatosis with polyangiitis.

ROC analyses were used to determine cutoff values of ferritin for systemic involvement and pulmonary hemorrhage. We showed two ROC curves of ferritin for predicting the systemic visceral involvement and pulmonary hemorrhage (Table 3). The AUCs of ferritin for systemic involvement and pulmonary hemorrhage were 0.85 (95%CI: 0.73–0.96, *p* < .001) and 0.94 (95%CI: 0.87–1.0, *p* < .001), respectively.

**Table 3.** Sensitivity, specificity, positive and negative predictive values of serum ferritin for the systemic involvement and pulmonary hemorrhage

Ferritin cutoff value	Sensitivity %	Specificity %	Positive predictive value %	Negative predictive value %
Systemic visceral disease				
300 μg/L	62.5	90	95.2	42.9
Pulmonary hemorrhage				
300 μg/L	92.9	71.4	61.9	95.2
550 μg/L	87.5	89.3	80.0	92.6

## Discussion

In this retrospective study, we found that serum ferritin was correlated well with the disease activity of GPA. Moreover, ferritin was remarkably elevated in severe visceral manifestations of disease. Our results suggest that serum ferritin could be a promising biomarker for the assessment of disease activity and potential predictive factor for the severe involvement of GPA.

Granulomatosis with polyangitis is a severe necrotizing vasculitis with high morbidity and mortality [18]. Disease- and treatment-related damages are common and relapse risk is as high as 50% [19]. Lack of markers that predict activity and relapses make management of GPA challenging and there is no clear consensus on the duration of maintenance treatment. Hence, reliable biomarkers are needed to guide the treatment [20]. Several studies have shown association between serum ferritin and disease severity in various inflammatory disorders including renal involvement in SLE and interstitial lung disease in dermatomyositis [13,14]. Therefore, some authors suggest use of more intensive treatment in newly diagnosed dermatomyositis patients with high serum ferritin levels [13].

Current studies show that immune cells, alternative complement pathway, adhesion molecules and several pro-inflammatory cytokines have roles in the pathogenesis of GPA. Briefly, ANCA produced from auto-reactive B cells activate primed neutrophils and activated neutrophils produce reactive oxygen species and release their lytic contents, initiating vasculitis. Even low doses of pro-inflammatory cytokines, including TNF-alpha, interleukin- (IL) 1 and IL-18, may fuel the inflammation by priming neutrophils and increase their surface expression of ANCA antigens [21].

Increased ferritin levels are suggested to be mediated by cytokines at transcriptional, post-transcriptional, and translational stages. These regulatory cytokines include IL-1, IL-6, IL-18 and TNF-alpha, all of which are also involved in the pathogenesis of GPA [22,23]. Therefore, increased ferritin levels could be explained by extreme cytokine production during acute stage of disease. Interestingly, in this study, there was no correlation between BVAS/WG and other acute phase reactants, but a significant correlation was demonstrated with serum ferritin levels. Whether ferritin contributed directly to the pathogenesis of severe manifestations of GPA as suggested in Still’s disease, remains to be elucidated.

There are several limitations in our study. Major drawback was its retrospective design. Serum ferritin level is known to be influenced by many factors including, infection, inflammation and blood transfusions. These patients were excluded from the study. Another limitation of the study is that we did not perform serial ferritin measurements; therefore, we could not determine whether its levels changed with treatment and may it predict relapse [24]. ANCA test was performed only with IFA method; therefore, serum ANCA titers were not availale for making correlation analyzes.

## Conclusions

Measurement of serum ferritin level at disease onset might help to stratify those patients who are at the highest risk for complications of GPA. Value of ferritin for the assessment of long-term prognosis and treatment response remain to be determined in longitudinal studies.
